# Biomechanical Study of a Tricompartmental Unloader Brace for Patellofemoral or Multicompartment Knee Osteoarthritis

**DOI:** 10.3389/fbioe.2020.604860

**Published:** 2021-01-28

**Authors:** Chris A. McGibbon, Scott Brandon, Emily L. Bishop, Chris Cowper-Smith, Edmund N. Biden

**Affiliations:** ^1^Faculty of Kinesiology and Institute of Biomedical Engineering, University of New Brunswick, Fredericton, NB, Canada; ^2^School of Engineering, University of Guelph, Guelph, ON, Canada; ^3^Department of Mechanical and Manufacturing Engineering, University of Calgary, Calgary, AB, Canada; ^4^Spring Loaded Technologies, Halifax, NS, Canada; ^5^Department of Mechanical Engineering and Institute of Biomedical Engineering, University of New Brunswick, Fredericton, NB, Canada

**Keywords:** knee brace, patellofemoral force, tibiofemoral force, simulation, osteoarthritis, tendon force, cruciate force, deep knee bend

## Abstract

**Objective:** Off-loader knee braces have traditionally focused on redistributing loads away from either the medial or lateral tibiofemoral (TF) compartments. In this article, we study the potential of a novel “tricompartment unloader” (TCU) knee brace intended to simultaneously unload both the patellofemoral (PF) and TF joints during knee flexion. Three different models of the TCU brace are evaluated for their potential to unload the knee joint.

**Methods:** A sagittal plane model of the knee was used to compute PF and TF contact forces, patellar and quadriceps tendon forces, and forces in the anterior and posterior cruciate ligaments during a deep knee bend (DKB) test using motion analysis data from eight participants. Forces were computed for the observed (no brace) and simulated braced conditions. A sensitivity and validity analysis was conducted to determine the valid output range for the model, and Statistical Parameter Mapping was used to quantify the effectual region of the different TCU brace models.

**Results:** PF and TF joint force calculations were valid between ~0 and 100 degrees of flexion. All three simulated brace models significantly (*p* < 0.001) reduced predicted knee joint loads (by 30–50%) across all structures, at knee flexion angles >~30 degrees during DKB.

**Conclusions:** The TCU brace is predicted to reduce PF and TF knee joint contact loads during weight-bearing activity requiring knee flexion angles between 30 and 100 degrees; this effect may be clinically beneficial for pain reduction or rehabilitation from common knee injuries or joint disorders. Future work is needed to assess the range of possible clinical and prophylactic benefits of the TCU brace.

## Introduction

Knee braces are a common conservative treatment option for reducing pain and improving function in people with musculoskeletal injuries and disease (Chew et al., 2007), such as knee osteoarthritis (OA) (Ramsey and Russell, 2009; Petersen et al., 2016; Phillips et al., 2016). Unicompartment off-loader braces are a common style of knee brace designed to reduce pain and progressive degeneration of the osteoarthritic knee by redistributing compressive forces away from the diseased medial or lateral tibiofemoral (TF) compartment via joint realignment (Ramsey et al., 2007; Briem and Ramsey, 2013). Although the use of these braces in patients with unicompartmental TF disease is supported by biomechanical (Moyer et al., 2015; Petersen et al., 2016) and clinical studies (Rannou et al., 2010; Mistry et al., 2018), the effectiveness of unicompartment off-loader braces may be limited because the vast majority (>90%) of patients have bicompartmental or tricompartmental disease with patellofemoral (PF) involvement (Duncan et al., 2009; Heekin and Fokin, 2014).

**Figure 1:**
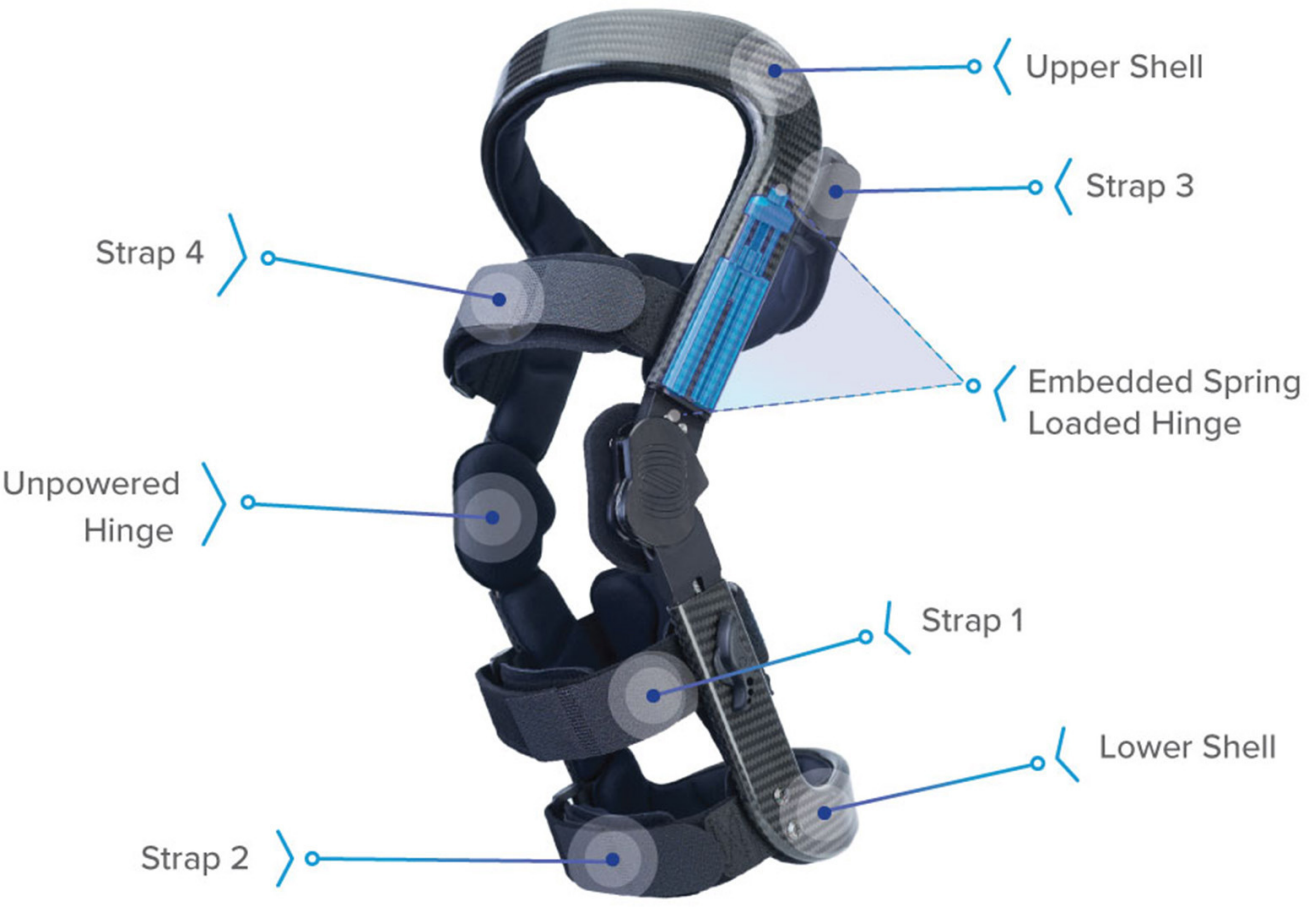
Levitation™ tricompartment unloader brace (Spring Loaded Technologies, Halifax, Nova Scotia, Canada). Components include straps to attach the brace to the leg, upper and lower carbon-fiber shells, and spring-powered and unpowered hinges. When the knee joint is flexed (e.g., during a squat), the spring-powered lateral hinge applies an assistive extension moment to reduce the user's quadriceps muscle effort. This assistive moment is transferred to the back of the user's leg via reaction forces at the proximal strap (Strap 3) and lower shell and cuff as an anteriorly directed force, ~20 cm above- and below-knee center (Budarick et al., 2020).

A new brace concept was recently introduced to provide simultaneous *unloading* (rather than *off-loading*) benefits to multiple knee compartments (i.e., TF and PF compartments) by reducing sagittal plane muscle effort (Budarick et al., 2020), with the goal of reducing pain and improving joint function for individuals with multicompartmental knee OA (Waller et al., 2011). The Levitation^®^ Tri-Compartment Unloader (Spring Loaded Technology Inc., Halifax, Nova Scotia, Canada), herein referred to as the tricompartment unloader (TCU) (Figure 1), is a passive mechanical brace capable of energy storage and body weight (BW) support during gravity assisted knee flexion (such as lowering to a squat). Stored energy is then returned to the user during antigravity motion (such as rising from a squat). There are currently three TCU brace models available from the manufacturer, which provide different levels of assistance across the range of motion of the brace. These brace models are designed for different therapeutic purposes or to meet varying user requirements. As muscles are the primary contributors to PF and TF joint contact loads that increase during weight-bearing flexion (Kuster, 2002; Winby et al., 2009; Sasaki and Neptune, 2010; Trepczynski et al., 2012), reduction in muscle effort should reduce the forces transmitted through the joint structures (Budarick et al., 2020). As *in vivo* measurements are generally infeasible, a model is required to quantify these effects.

There are a wide variety of models in literature that can be used to estimate changes in knee loads due to an assistive brace [see review in Fregly et al. (2012)]. On one end of the spectrum, finite element models provide high-resolution estimates of tissue-level stress and strain but at high computational cost (Kazemi et al., 2013; Shriram et al., 2019). Conversely, reductionist models (Morrison, 1968; Wismans et al., 1980; Yamaguchi and Zajac, 1989) rely on simplifying assumptions to lump or omit some tissues, but may still be of sufficient quality to reveal the net action of load-bearing structures of the knee (Dumas et al., 2019) and to estimate the unloading effect of a knee brace (Pollo et al., 2002; Budarick et al., 2020). Regardless of the modeling approach, validation is both crucial and challenging (Hicks et al., 2015).

Because the TCU brace acts primarily in the sagittal plane, in the present study we chose a planar model of the knee previously described by O'Connor et al. (1989) and expanded upon in several studies (Collins and O'Connor, 1991; Zavatsky and O'Connor, 1992a,b; Lu and O'Connor, 1996; Wilson et al., 1998; Huss et al., 2000; Imran et al., 2000). The model is based on the crossed four-bar geometry of the anterior and posterior cruciate ligaments (ACL and PCL, respectively), which governs TF and PF contact kinematics. When coupled with external loads from foot–floor reactions, limb inertia and gravity, and forces applied from an external source (such as a brace or other orthotic), this model can readily compute forces in the extensor (quadriceps) or flexor (hamstrings) tendons, the ACL or PCL, and TF and PF contact forces.

The main objective of this study was to use the model, with kinematic and kinetic motion analysis data from healthy participants, to simulate the biomechanical effects of wearing the different TCU brace models during a weight-bearing deep knee flexion activity. The deep knee bend (DKB) test is commonly used in clinical studies of the knee (Stiehl et al., 2001; Komistek et al., 2003; Argenson et al., 2004) as it is known to increase knee joint force (Kutzner et al., 2013) and often results in increased pain and/or dysfunction in patients with knee OA (Wijayaratne et al., 2007).

Because the intended function of the TCU brace is to reduce TF and PF contact forces during weight-bearing knee flexion, it is important to be confident in the predictions of the model. The secondary objective was therefore to establish the fidelity of the model predictions. To this end, we evaluated the sensitivity of the force predictions to uncertainties in knee model geometry and validated the model output against a criterion standard, in this case the Grand Challenge (GC) data from Fregly et al. (Fregly et al., 2012), as well as other published literature. This allowed us to define a valid range within which to compare the simulated braced condition for the different TCU brace models with the non-braced condition, and make conclusions about TCU unloading behavior.

## Materials and Methods

### Motion Analysis Dataset

Three-dimensional (3D) human movement data for healthy participants who participated in a different study (Mohamed et al., 2019) were used in this simulation study. Data from fourteen participants who performed a DKB test were screened for inclusion in this study, requiring that peak knee flexion angle during the DKB task was ≤ 135 degrees, due to constraints of the knee model (O'Connor et al., 1989). This resulted in a sample of eight participants (age 25 ± 5 years, mass 66 ± 12 kg, height 164 ± 8 cm, six females). The DKB test protocol is detailed elsewhere (Mohamed et al., 2012). Briefly, participants stood with each foot on side-by-side force plates and completed the DKB task three times. All participants provided informed consent for the original study, and the present secondary analysis of their data was approved by the university research ethics board.

### Biomechanical Knee Model

#### Lower Leg and Knee Model

**Figure 2:**
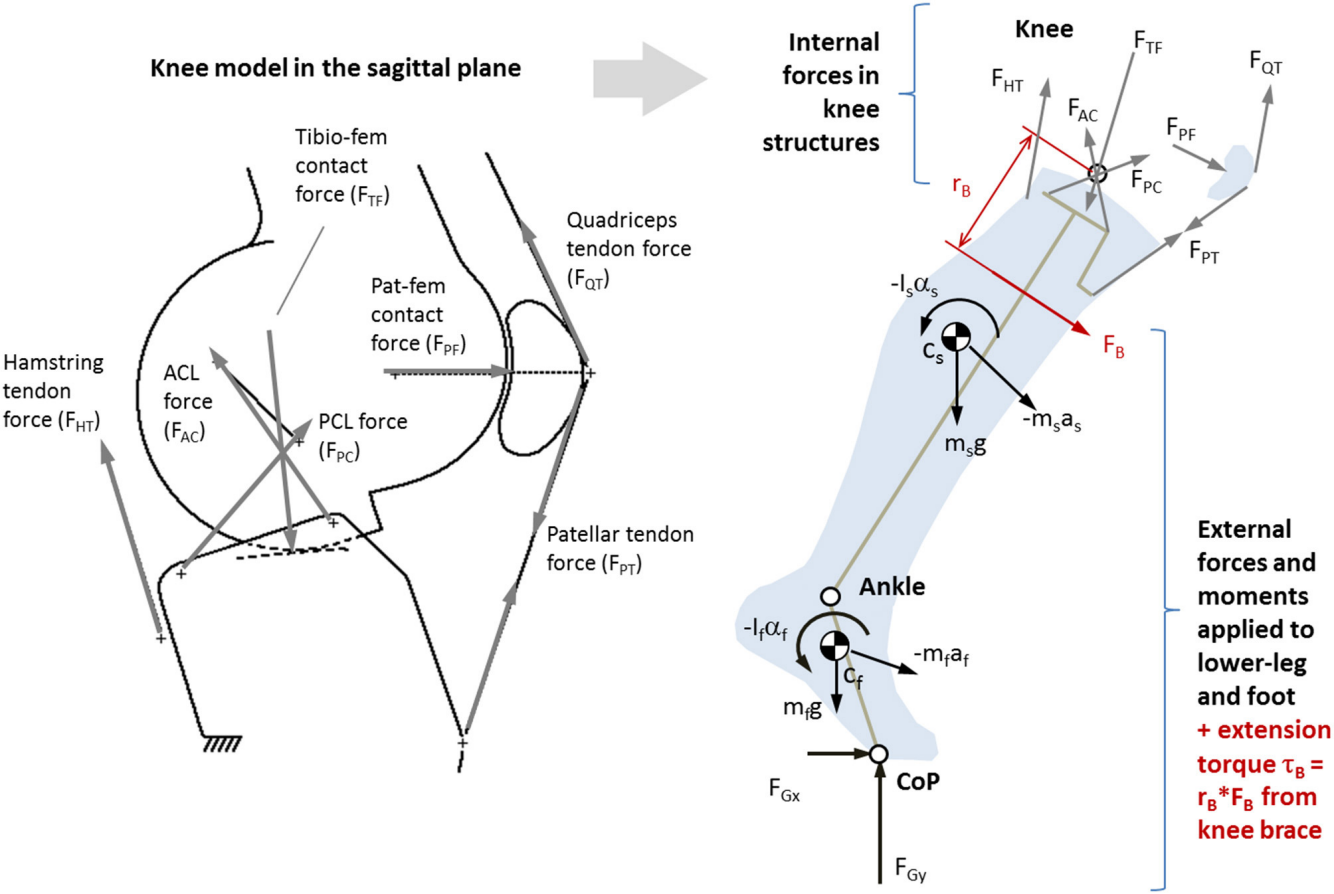
Left: Single degrees-of-freedom kinematic knee model used to determine position and orientation of major load-bearing knee structures (O'Connor et al., 1990a). Right: Sagittal plane (2D) biomechanical model of the lower-leg and knee based on O'Connor et al. (1990b), where ground forces and segmental kinematics come from 3D gait analysis. Also shown is a moment of force caused by an external source (such as an active brace) *F*_B_ acting with moment arm *r*_B_.

A 3D inverse dynamics model of the lower-leg and foot (McGibbon et al., 2017) was used to compute net forces at the knee joint. Then, a sagittal plane model of the knee (O'Connor et al., 1989), as shown in Figure 2, was used to resolve muscle tendon forces, cruciate ligament forces, and joint contact forces in the sagittal plane. Model geometry (see Supplementary Material Appendix I, Table A1) was scaled to each participant's tibia length.

The knee kinematic model, first described by Kapandji (1987) and later expanded by O'Connor and colleagues (O'Connor et al., 1989; Collins and O'Connor, 1991; Zavatsky and O'Connor, 1992a,b; Lu and O'Connor, 1996; Wilson et al., 1998; Huss et al., 2000; Imran et al., 2000), treats the cruciate ligaments as having a theoretical isometric fiber that, in combination with the TF contact surfaces, can be modeled as a crossed four-bar linkage where the moving (femoral) condyle rolls and slides on the fixed (tibia) plateau as a function of knee flexion angle. The knee flexor and extensor mechanisms were modeled as described by O'Connor et al. (1990a,b). The kinematic model is described in detail in Supplementary Material Appendix I.

Given a prescribed knee flexion angle, the kinematic model outputs the origin and orientation of the ACL and PCL lines of action (LoA), TF contact LoA, hamstring tendon LoA, and LoA of the quadriceps mechanisms (patellar tendon, quadriceps muscle tendon, and PF contact). As such, the force system at the proximal tibia (Figure 2) is underdetermined with six unknown forces to achieve dynamic equilibrium: hamstring tendon force (*F*_HT_), patellar tendon force (*F*_PT_), anterior cruciate force (*F*_AC_), posterior cruciate force (*F*_PC_), and TF contact force (*F*_TF_). At each instant in time, the system was reduced into four fully determined candidate solutions, each consisting of the only three forces (*F*_TF_, plus one cruciate and one tendon force). The candidate solution yielding a tensile (positive) ligament and tendon force, and lowest compressive (negative) contact force, was retained (see Supplementary Material Appendix for details). If the patellar tendon force was non-zero, PF contact force (F_PF_) and quadriceps tendon force (*F*_QT_) were determined by treating the patella as a 3-force body.

#### Sensitivity and Validity

Because this was a retrospective simulation where participants' data did not include detailed quantification of subject-specific knee geometry (e.g., medical imaging), it was important to first evaluate the sensitivity of the model to uncertainty in knee geometry input parameters. Therefore, a parameter analysis was conducted using estimated uncertainties of each input parameters of the knee geometry model. Uncertainties were assumed to be δ*v* +/– 5 mm in linear dimensions and δ*v* +/– 5 degrees in angular dimensions (see Table A.1 for details). These values were based on measurement precision of joint tissue attachments from magnetic resonance imaging (MRI) studies (Bosmans et al., 2015).

In this analysis, inertial contributions (mass, mass moment of inertia, and limb accelerations) and ground reaction forces were assumed true. An expanded Taylor's series was used to combine the parametric output uncertainties (δ*F*_υ*i*_), based on the maximum likelihood estimator:

(1) $$\begin{array}{l} \delta F\;=\;\sqrt{\frac{\left(\sum_{\nu i=1}^{M}\delta F_{vi}^{2}\right)}{M}} \end{array}$$

where *M* is the number of variables included in the sensitivity analysis, where *M* = 14 in this study.

In order to determine which parameter uncertainties were most influential, each parameter's contribution (ε_υi_) to the total uncertainty was expressed by rearranging the above equation and normalizing the left side to unity by dividing parameter squared uncertainties by total squared uncertainty.

(2) $$\begin{array}{l} 1=\left(\delta F_{\nu 1}^{2}+\delta F_{\nu 2}^{2}+\cdots +\delta F_{\nu M}^{2}\right)/\left(\delta F^{2}M\right)=\varepsilon_{\nu 1}+\varepsilon_{\nu 2}+\cdots /\\ \;+\varepsilon_{\nu M} \end{array}$$

or

(3) $$\begin{array}{l} \varepsilon_{\nu }1=\delta F_{\nu 1}^{2}/\left(\delta F^{2}M\right);\;\varepsilon_{\nu }2=\delta F_{\upsilon 2}^{2}/\left(\delta F^{2}M\right);\;\cdots \;;\;\varepsilon_{\nu M}\\ \;=\delta F_{\nu M}^{2}/\left(\delta F^{2}M\right) \end{array}$$

In order to evaluate the overall sensitivity of the force outputs to input geometry uncertainties, the “variability ratio” of mean sum of square errors δ*F*^2^ to between-subject variability σ_*F*_^2^ (variance of force across participants) was computed.

(4) $$\begin{array}{l} \upsilon =\frac{\delta F^{2}}{\sigma_{F}^{2}} \end{array}$$

A variability ratio υ << 1 would indicate that parameter sensitivity is less a concern than natural between-subject variability, whereas υ approaching or greater than unity would suggest model results may not be trusted. We arbitrarily selected a ratio of 20% (υ = 0.2) as the threshold of acceptability.

Finally, to determine the range in which the knee model is valid, a two-legged DKB activity was simulated using published motion capture data from *N* = 4 participants with instrumented TKR from the 3rd (male, mass = 70 kg, height = 172 cm), 4th (male, 75 kg, 180 cm), 5th (male, 66.7 kg, 168 cm), and 6th (female, 78.4 kg, 167 cm) GC competitions to predict *in vivo* knee forces (Fregly et al., 2012). Model predictions of TF forces were compared with synchronized *in vivo* measurements using root mean square error (RMSE) and reported as a function of knee angle. These data were used to specify the knee flexion range where the model sufficiently agrees (95% confidence interval) with *in vivo* measured forces.

### TCU Brace Simulation

#### Simulation

**Figure 3:**
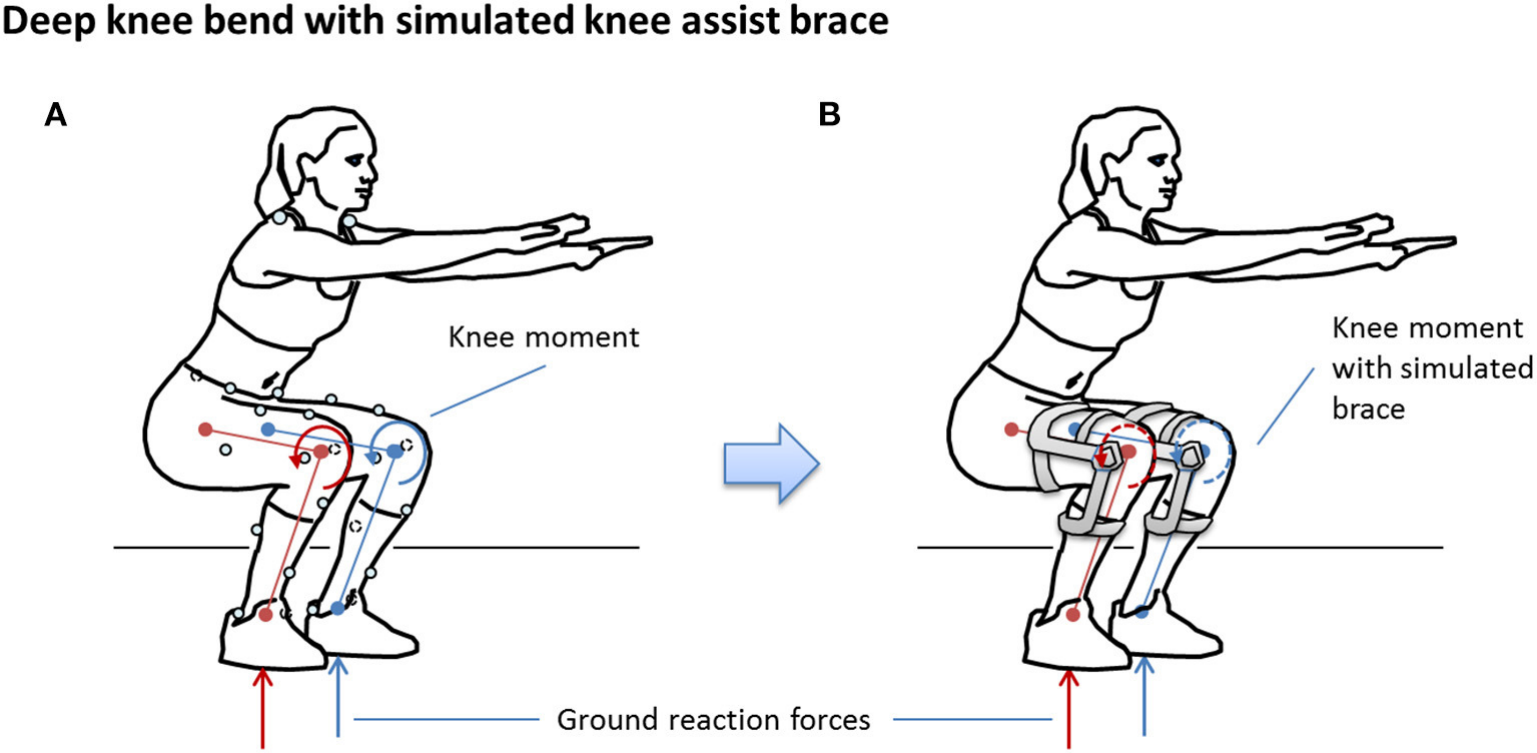
Deep knee bend test: **(A)** observed test without brace, **(B)** simulated test with brace.

The simulation was predicated on the assumption that participants could, in theory, produce the same kinematics and ground reaction forces as the non-braced condition for each of the simulated brace conditions (Brandon et al., 2019), as illustrated in Figure 3. In this way, the simulation can be used to study the response of knee tissue forces for different TCU brace models relative to the no-brace condition to provide a scientific justification for advancing to human trials of the brace.

The design of the TCU brace is detailed elsewhere (Budarick et al., 2020). Briefly, the brace is coupled to the upper and lower leg in a similar manner to traditional rigid frame knee braces. Unlike traditional knee braces, the lateral hinge contains a powerful liquid spring to absorb BW and assist the extensor mechanism in the sagittal plane by applying an external torque to the knee, via a force couple created by the cuff contact points on the leg. For the purpose of a ground-up analytical approach, the brace extension assist moment is transferred to the back of the lower leg ~20 cm below the knee center. Brace moment arm *r*_B_ was therefore assumed to be fixed at 20 cm.

**Figure 4:**
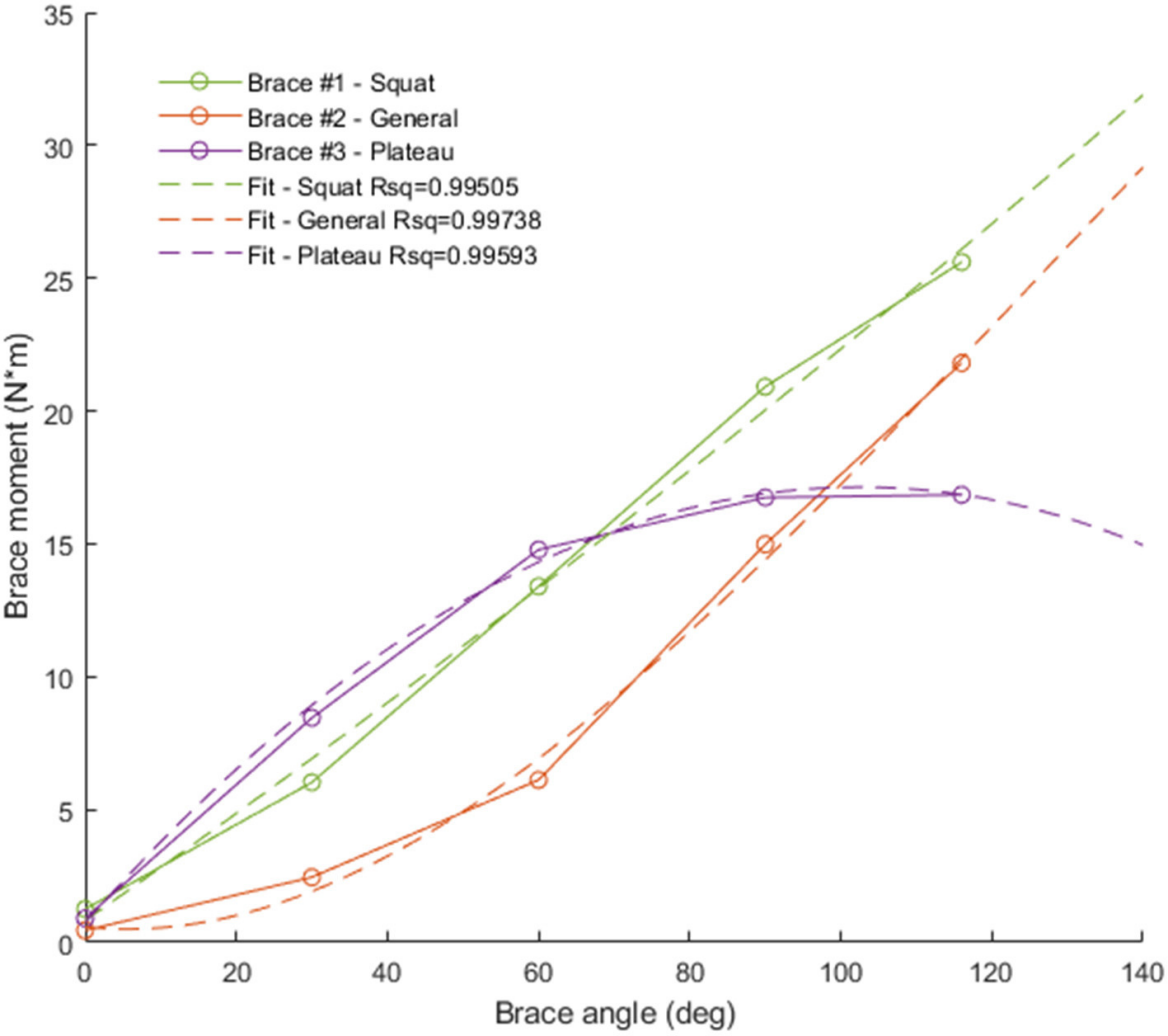
Brace moment/angle curves used in the simulation. Green = squat brace (brace #1, linear fit), red = general brace (brace #2, quadratic fit), purple = plateau brace (brace #3, quadratic fit).

Three different TCU brace models (squat, plateau, and general) were considered in this study (Table 1). The corresponding brace moment/angle calibration curves were determined by the manufacturer using bench experiments, as described in (Budarick et al., 2020) (Figure 4). For this proof-of-concept study, the brace was modeled as a force *F*_B_ applied perpendicular to the tibia located distance *r*_B_ below the knee. *F*_B_ magnitude was calculated from the moment/knee flexion curve using a look-up table where the input was knee angle, and output was brace force (moment divided by 0.02 m) in Newtons (N).

**Table 1:** Simulated brace conditions.

**Brace condition**	**Description**
No brace	Brace moment is 0 at all knee angles (observed case).
Brace model 1–squat	Brace moment increases with knee angle in an approximate linear fashion. This brace model should provide the greatest amount of knee assist throughout the range of knee motion of a squat.
Brace model 2–general	Brace moment increases with knee angle gradually at first and with increasing force at higher flexion angles. This brace model was designed for general purpose use.
Brace model 3–plateau	Brace moment increases with knee angle but plateaus at ~100–110 degrees of knee flexion. This brace model supports the knee at lower flexion angles.

#### Statistical Analysis

We compared non-brace and brace conditions using Statistical Parameter Mapping (SPM), a relatively recent waveform analysis technique. Detailed description of this analysis technique can be found elsewhere (Kiebel and Friston, 2004; Pataky et al., 2014, 2016). Briefly, SPM operates analogous to standard *t*-test and analysis-of-variance tests but considers the whole (or part of the) waveform for comparison, identifying the region where the waveforms significantly (at a selected α level) depart from one another. We denote this region as the “effectual region” of the brace assist. The effectual region was thus defined by the region where the continuum of *t* scores across the waveform comparison exceeded the critical *t* score (t^*^) at the selected α level. We used the SPM analog of the paired *t*-test, with Bonferroni α correction to account for multiple comparisons of the three braced conditions to the non-braced condition (α = 0.067). For ease of interpretation, we also compared peak forces at an arbitrary knee flexion angle of 90 degrees using paired *t*-tests (α = 0.067), which represents a reasonably deep squat within the valid range of our model.

## Results

### Biomechanical Knee Model

**Figure 5:**
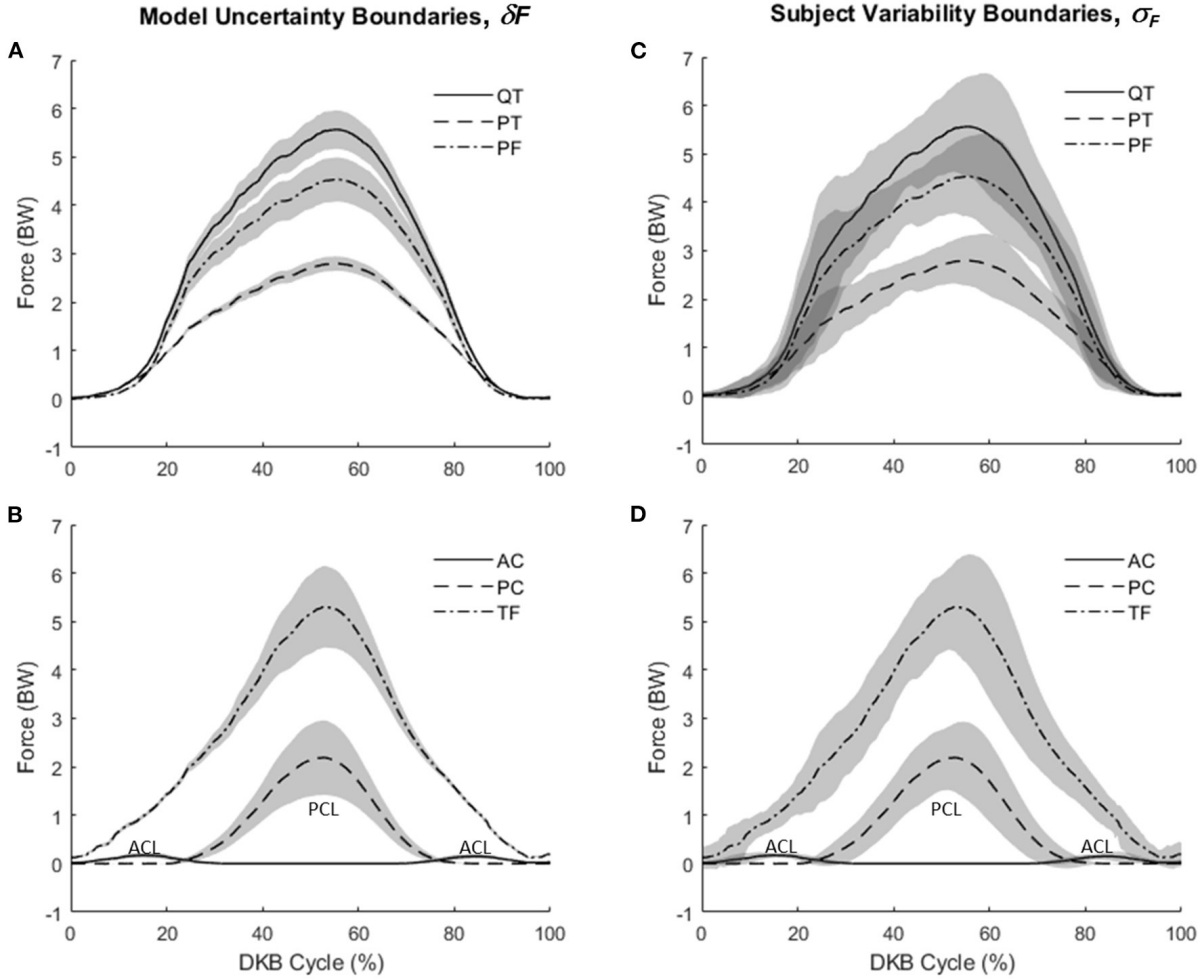
Knee forces during deep knee bend (DKB) using the model (no-brace condition). **(A)** Mean values for patellar tendon (PT), quadriceps tendon (QT), and patella–femoral contact (PF) with shaded regions showing *model* uncertainty (+/–δ*F*). **(B)** Mean values for anterior cruciate (AC), posterior cruciate (PC), and tibiofemoral contact (TF) with shaded regions showing *model* uncertainty (+/–δ*F*). **(C)** Mean values for patellar tendon (PT), quadriceps tendon (QT), and patella–femoral contact (PF) with shaded regions showing *subject* variability (+/–σ_*F*_). **(D)** Mean values for anterior cruciate (AC), posterior cruciate (PC), and tibiofemoral contact (TF) with shaded regions showing *subject* variability (+/–σ_*F*_).

Mean knee forces during DKB are shown in Figure 5. Figures 5A,C show QT, PT, and PF forces, whereas Figures 5B,D show AC, PC, and TF forces. Extensor mechanism forces were highest for QT followed by PF and PT forces. For the cruciate–TF complex, forces were highest for the TF contact force, followed by the PC and AC ligaments. The AC ligament was only loaded in the early and late portions of the DKB, with the PC ligament supporting the shear force across the knee during midportion of the DKB. As shown by the shaded boundaries, subject variability (Figures 5C,D) was higher than model uncertainty (Figures 5A,B), except toward the midportion of DKB.

**Figure 6:**
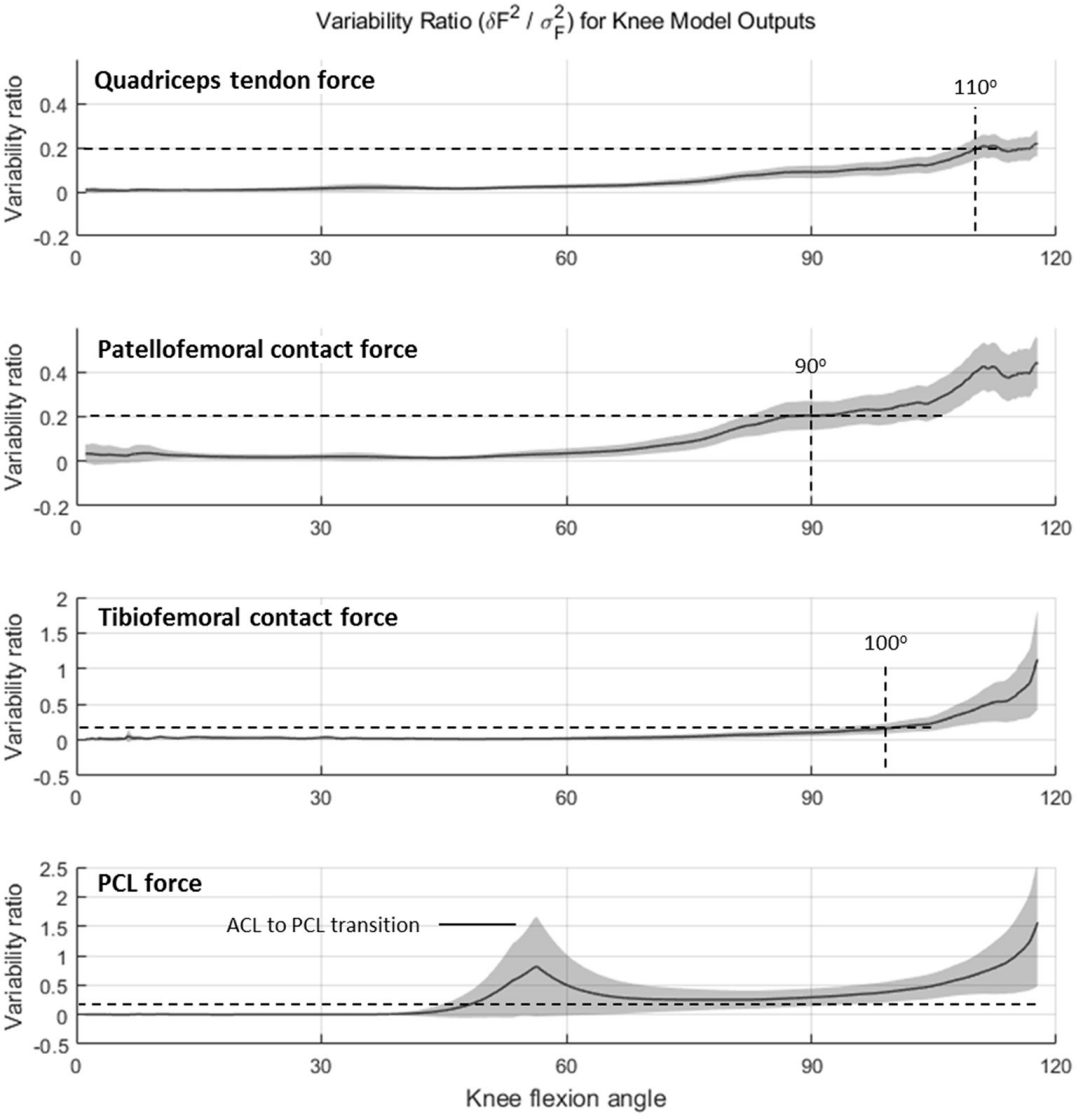
Variability ratio (mean sum of square errors δ*F*^2^ divided by between-subject variance σ_*F*_^2^) in knee force estimates over the range of knee flexion during the deep knee bend (DKB) task for quadriceps tendon, patella–femoral contact, tibiofemoral contact and PCL (no-brace condition).

Variability ratio (υ) using Equation (4) is plotted as a function of knee flexion angle (for the DKB descent phase) in Figure 6. For QT force, TF, and PF contact forces, the sensitivity of the model to geometric parameters is negligible in comparison with between-subject variability up until ~60 degrees of knee flexion, after which it starts to increase. The dashed horizontal projection lines at a variability threshold of υ = 0.2 are shown intersecting the curve at knee angles of 90 degrees or higher for QT, PF, and TF forces. The PC ligament force, however, remained (when active) above this threshold after 50 degrees and reached unity at ~115 degrees.

**Figure 7:**
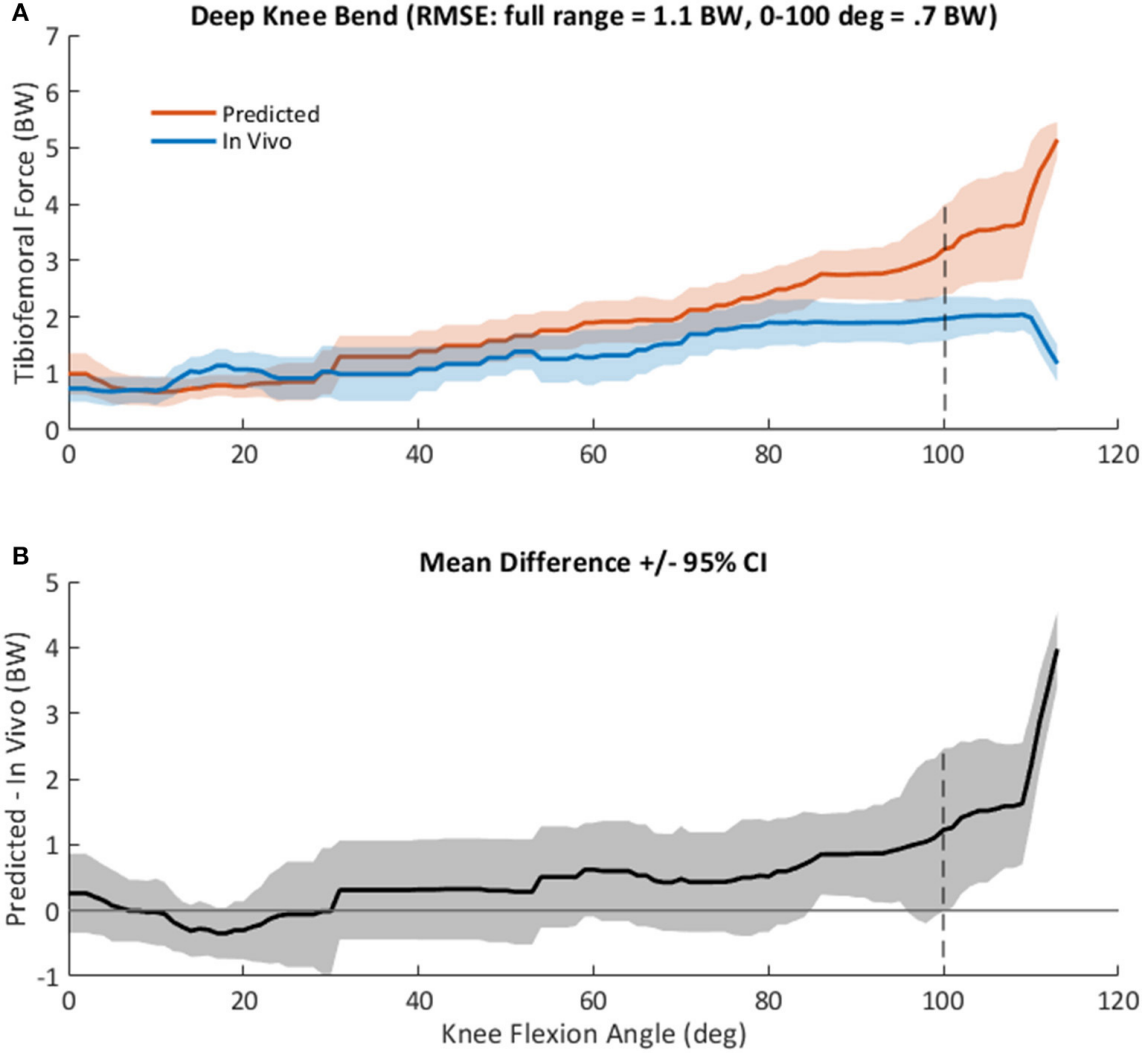
**(A)**, Mean +/– standard deviation (SD) tibiofemoral contact force, *F*_TF_, as predicted by the computational knee model (red), and as measured *in vivo* from the instrumented tibial implant (blue) in four subjects during a squat activity (Winby et al., 2009). Root mean square prediction error was 1.1 times body weight (BW) across the entire range, but only 0.7 BW at knee angles <100 degrees. **(B)**, Mean +/– 95% confidence interval of the difference between predicted and measured knee loads, as shown in **(A)** vs. knee flexion angle across all subjects.

Evaluation of the model using data from four participants in the GC dataset is summarized in Figure 7. Model predictions of TF contact force followed the general pattern of *in vivo* measurements, increasing with knee flexion angle and reaching ~3.5 BW at 100 degrees of knee flexion (Figure 7A). RMSE prediction error was 0.7 BW between 0 and 100 degrees of knee flexion, but larger (1.1 BW) across the entire DKB motion. Predictions agreed with *in vivo* measurements (i.e., 95% confidence interval included 0) until ~100 degrees of knee flexion, with a bias toward overestimation (Figure 7B). The largest prediction error (>2 BW) was observed at high flexion angles (>110 degrees), which were achieved by only one of the four total knee arthroplasty (TKA) participants.

Both the sensitivity analysis and the validity analysis indicate that the region of knee flexion where joint forces from the analytical knee model may be considered trustworthy is ~0 to 100 degrees of knee flexion.

### TCU Brace Simulation

**Figure 8:**
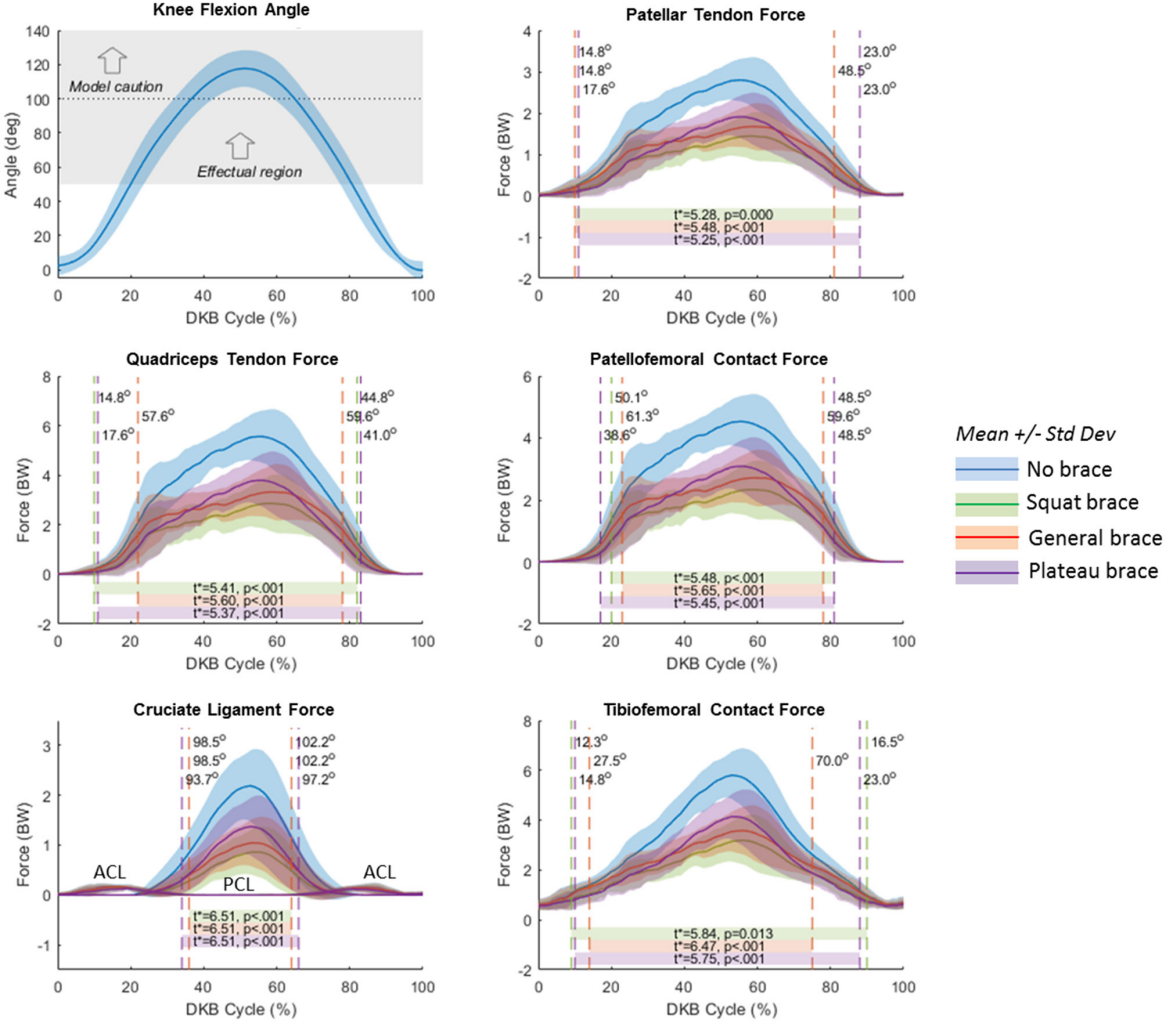
Statistical Parameter Mapping (SPM) results for no-brace condition compared to each simulated brace condition (moment/angle curve). Blue curves represent the no-brace condition. Green represents the squat brace; red represents the general brace, and purple represents the plateau brace. Horizontal bars represent the SPM predicted effectual region where the force curves of each brace condition are significantly different than the no-brace condition. Vertical dashed lines represent the point at which the calculated *t* scores exceeded the critical t*, and labels indicate the corresponding knee flexion angles at the t* threshold crossings. The top left plot shows knee flexion angle with shaded region illustrating the average effectual region of the brace (>50-degree knee flexion), and the horizontal dotted line representing the limit above which the model results should be interpreted with caution.

SPM analysis results are summarized in Figure 8. Effectual regions (α = 0.067) of the non-braced condition vs. each of the braced conditions (squat, general, and plateau) are shown by horizontal bars at the bottom of each plot. These are the regions where the braced condition resulted in significant reduction of knee tissue force relative to the non-braced condition. Vertical dashed lines show the t^*^ threshold crossings and the corresponding knee flexion angle (shown in Figure 8, top left) at which they occur. Text inside the horizontal bars shows the critical t^*^ values and corresponding *p* values. The effectual range of the squat and plateau brace models occurred at knee angles >15–20 degrees for PT, QT, and TF forces; >40 degrees for PF force; and >95 degrees for PC ligament force. Effectual range for the general brace model was consistently smaller than for the squat and plateau brace models; for example, the general brace model required knee angles >50 degrees of flexion to reduce the PF contact force.

Mean knee forces at 90 degrees of flexion during descent and ascent portions of the DKB are summarized in Table 2 for the non-braced condition and each of the three simulated braced conditions.

**Table 2:** Knee forces at 90 degrees of knee flexion [units of body weight (BW)] during observed DKB descent and ascent (non-braced) and with three different simulated TCU brace models for healthy participants (*n* = 8).

**Force in units of BW**	**No brace**		**Squat brace**		**Effect**		**Gen. brace**		**Effect**		**Plat. brace**		**Effect**	
	**Mean**	**SD**	**Mean**	**SD**	***p***	***d***	**Mean**	**SD**	***p***	***d***	**Mean**	**SD**	***p***	***d***
Descent														
*F*_PT_	2.07	0.35	1.10	0.32	<0.001	4.31	1.37	0.31	<0.001	4.32	1.26	0.32	<0.001	4.30
*F*_PF_	3.46	0.59	1.84	0.54	<0.001	4.31	2.29	0.52	<0.001	4.32	2.10	0.53	<0.001	4.30
*F*_QT_	4.13	0.70	2.19	0.64	<0.001	4.31	2.73	0.63	<0.001	4.33	2.51	0.63	<0.001	4.30
*F*_TF_	2.96	0.47	1.66	0.40	<0.001	4.27	2.01	0.41	<0.001	4.37	1.86	0.41	<0.001	4.29
*F*_AC_	0.00	0.00	0.02	0.02	0.049	−0.84	0.01	0.01	0.351	−0.35	0.01	0.02	0.281	−0.41
*F*_PC_	0.50	0.15	0.16	0.11	<0.001	4.66	0.23	0.13	<0.001	4.12	0.20	0.12	<0.001	4.24
Ascent														
*F*_PT_	2.10	0.28	1.12	0.24	<0.001	4.27	1.39	0.23	<0.001	4.27	1.28	0.23	<0.001	4.28
*F*_PF_	3.50	0.47	1.88	0.39	<0.001	4.28	2.33	0.38	<0.001	4.27	2.14	0.39	<0.001	4.29
*F*_QT_	4.19	0.57	2.24	0.47	<0.001	4.27	2.78	0.46	<0.001	4.27	2.56	0.46	<0.001	4.28
*F*_TF_	2.97	0.41	1.67	0.33	<0.001	4.74	2.02	0.34	<0.001	4.47	1.87	0.34	<0.001	4.59
*F*_AC_	0.00	0.00	0.01	0.03	0.148	−0.57	0.00	0.00	—	—	0.01	0.01	0.179	−0.53
*F*_PC_	0.48	0.11	0.13	0.09	<0.001	7.23	0.21	0.11	<0.001	5.09	0.19	0.10	<0.001	7.00

*SD = standard deviation, p = significance value (at Bonferroni corrected α = 0.003), d = Cohen d (effect size), F_PT_ = patellar tendon force, F_PF_ = patellofemoral contact force, F_QT_ = quadriceps tendon force, F_TF_ = Tibiofemoral contact force, F_AC_ = ACL force, F_PC_ = PCL force. Effect for each brace is relative to the no-brace condition*.

Knee forces were significantly (*p* < 0.001) reduced for all three simulated brace models compared to the observed non-braced condition. Effect sizes were large (*d* > 4, Table 2) for all joint structures except the AC ligament, which is not loaded throughout most of the DKB (Figure 5B). The squat brace provided the largest unloading effect across all structures (>43% reduction), followed by the plateau brace (>37% reduction) and the general brace (>32% reduction). For the squat brace, the PC ligament force was most reduced (68% reduction), followed by PT, PF, and QT forces (46% reduction) and TF contact (43% reduction). Note, however, that the reduction of PC ligament forces reported should be interpreted with caution because of observed PC force sensitivity to modeling assumptions (Figure 6).

## Discussion

Unloading of joint contact forces is widely recognized as a best practice in the conservative care of knee OA patients and may be achieved through a range of strategies including BW reduction, activity modification, strengthening/exercise, and the use of walking aids or knee braces (Sarzi-Puttini et al., 2005; Zhang et al., 2008; Waller et al., 2011; Messier et al., 2018; Mistry et al., 2018). Joint unloading can result in clinically significant improvements in pain, function, and quality of life (Waller et al., 2011; Messier et al., 2018; Mistry et al., 2018) and may be used as a strategy to delay or prevent surgery (Lee et al., 2017) or even slow the progression of knee OA (Radin and Burr, 1984; Block and Shakoor, 2009; Mastbergen et al., 2013; van der Woude et al., 2017). This study analyzed the biomechanics of the knee joint for three different models of a novel TCU brace, which is designed to reduce joint forces in all three knee compartments when the knee is flexed and weight-bearing.

Compared to the non-braced condition, significant force reduction was predicted for all major structures of the knee during the DKB task for the simulated TCU braced conditions. Because of its large assistive moment (Figure 4), the squat brace model provided the highest reduction in force, followed by the plateau brace and then the general brace. The squat and plateau braces were effectual (i.e., provided a significant reduction in force) beyond 20 degrees of knee flexion for TF and QT forces and beyond 50 degrees of knee flexion for PF forces. This result demonstrates multicompartment unloading beyond 50 degrees of knee flexion with the squat and plateau braces during the DKB. More specifically, QT, PT, PF, and TF forces were reduced by >40% with the squat brace at 90 degrees of flexion compared to the non-braced condition. In comparison, the general brace was effectual at reducing PF and QT forces beyond 60 degrees of knee flexion, and TF forces were significantly reduced beyond 70 degrees of knee flexion. Therefore, the general brace may also provide multicompartment unloading (~30% reduction); however, higher knee flexion angles are required to achieve this in comparison to the squat and plateau braces.

It was also apparent that the effectual region was not symmetric with respect to descent and ascent phases. As shown in Figure 8, the limit of the effectual region for each brace model occurred at a smaller knee flexion angle during descent than ascent. As the brace moment/angle curves were functions of flexion angle alone, without hysteresis, this differential performance between descent and ascent (see also Table 2) is attributed to biomechanical (or inertial) differences in each phase of the DKB test. Indeed, while the knee flexion angle was relatively symmetrical in time with a peak near 50% of the cycle, the joint tissue loads (QT, PT, PF; Figures 5, 8) exhibited skewed curves with much later peaks (~60% cycle). It is likely this asymmetrical behavior would also be present in the real-life behavior of the brace and therefore should be taken into account when interpreting the data.

### Mechanism of Joint Unloading With the TCU

The observed reduction in QT force with all three brace models suggests reduced sagittal plane muscle effort with TCU brace use. This provides evidence in support of the proposed mechanism of unloading, whereby reduced quadriceps muscle effort is expected to correspond with reduced force in knee joint structures. The current findings show significant reductions in both TF and PF joint contact forces with all three brace models, demonstrating that the TCU brace is capable of providing simultaneous unloading benefits to multiple compartments in the knee. Although our study was planar and therefore unable to quantify force sharing between medial and lateral TF compartments, given the mechanism of unloading (reduced QT and PT forces), it can be reasonably expected that both medial and lateral compartments would experience unloading during the DKB.

This finding differentiates the TCU from traditional unicompartment off-loader braces that are restricted to providing an unloading effect to one side of the TF joint by redistributing (or “off-loading”) forces to the opposite side of the knee (Gross and Hillstrom, 2008; Ramsey and Russell, 2009; Gohal et al., 2018; Budarick et al., 2020), an effect that could contribute to the development of bicompartmental OA (Gross and Hillstrom, 2008). By unloading all three compartments of the knee joint, the TCU may overcome these limitations while offering patients with PF or multicompartmental disease a bracing option that better matches their pattern of OA. The reduction in joint force achieved with the TCU is expected to decrease knee pain associated with OA that results from excessive joint loading (Felson, 2005). Importantly, we found no evidence of significant force increases in any knee tissue structures during the DKB task. This suggests that the TCU brace is safe to use during knee bend activity and should be tested with human participants to determine the effect on knee joint unloading across a range of activities.

The TCU may be of particular interest in the treatment of PF disorders resulting in PF pain such as trochlear dysplasia, chondromalacia, patellar tendonitis, and OA. These PF conditions are considered challenging to treat (McCarthy and Strickland, 2013), and conservative treatment with existing bracing technology that aims to realign the joint has not proven clinically beneficial (Hunter et al., 2011). The TCU brace may also be beneficial during rehabilitation from common knee injuries or surgical procedures. Joint unloading is believed to be beneficial for soft-tissue repair (van der Woude et al., 2017), and progressive weight bearing intended to gradually introduce joint loading is often applied during rehabilitation from meniscal (Cavanaugh and Killian, 2012), cartilage (Mithoefer et al., 2012), ligament (Cavanaugh and Powers, 2017), and tibial plateau fracture repair (Arnold et al., 2017) procedures. Unloading may also have benefits prophylactically, for example, to help prevent the development of knee OA or the occurrence of joint-overuse and joint-stress injuries (Takeda et al., 2011), which may be relevant from an occupational health and safety perspective. Future clinical outcomes research is required to further characterize these and other potential benefits.

### Performance of the Knee Model

The knee model results were largely consistent with results from other knee models evaluated during squat or DKB, in terms of the relative magnitudes of the QT, PT and PF forces (Nisell et al., 1986; Yamaguchi and Zajac, 1989), the transition between AC and PC ligament loading (Beynnon et al., 1996), and the magnitude of TF contact force (Shelburne and Pandy, 2002; Smith et al., 2008). The results of the present investigation also extend the findings of a prior study that calculated the theoretical unloading effect of the TCU at a static 90° knee bend (Budarick et al., 2020). Briefly, when outlining the design intent for the brace, Budarick et al. (Budarick et al., 2020) calculated that the squat TCU brace model would reduce contact forces of the PF and TF joints by ~22% at a 90° knee bend. The results of the present study indicated a larger reduction (>30–50%). The difference is explained at least in part by the volunteers that participated in both studies. In the study by Budarick et al. (2020), calculations were based on a single male participant with body mass of 93 kg. In the present study, average body mass of our participants (mostly female) was 66 kg. As the TCU brace applies the same assistive moment regardless of BW, it makes sense that our force reduction estimate is greater than that estimated by Budarick's analysis.

Overall, the knee model performed well under small (+/−5 mm or +/−5 degrees) perturbations in the geometric inputs. Figures 5, 6 show that between-subject variability in knee force estimates was greater than the variability in knee forces due to model input errors, up until ~90–100 degrees of knee flexion, after which input error influences growth rapidly, similar to that reported by Schellenberg et al. (Schellenberg et al., 2018). On the one hand, these findings suggest that a planar knee model with simple geometric scaling may be sufficient for calculating knee forces during activities having less extreme ranges. On the other hand, our results show that biomechanical analysis of the knee at more extreme flexion angles may require more precise measurement of geometric inputs (e.g., coregistered medical imaging, etc.) and/or a more sophisticated model (e.g., allowing for deformation of ligaments, etc.).

Lower limb bone geometry can be measured with very high accuracy using medical imaging techniques (Van den Broeck et al., 2014). *In vivo* measurement of cruciate ligament lengths and identification of attachment sites are a greater challenge, but promising results have been published using MRI and computed tomography (Rachmat et al., 2014; Lee et al., 2015), and efforts to automate the segmentation of soft tissues using deep learning approaches may result in more robust solutions (Tack et al., 2018; Mallya et al., 2019). Nevertheless, the capacity to acquire such measures routinely through medical imaging is rare. Therefore, our sensitivity analysis should provide some confidence to biomechanical researchers that linear cruciate–TF complex scaling based on anatomical length of the tibia is a good first approximation for the purposes of estimating joint force.

This conclusion was also borne out by the GC analysis, which showed acceptable agreement (RMSE = 0.7 BW) between instrumented and calculated TF force until ~100 degrees of flexion (or 1.1 BW across the entire range of movement), with mean prediction errors centered near zero (Figure 7). By comparison, in one recent review comparing various state-of-the-art modeling approaches, RMSE ranged from 0.3 to 0.88 BW vs. *in vivo* TF knee contact measurements during normal gait (Moissenet et al., 2017). Our study was also in general agreement with instrumented knee prosthesis studies (Mizu-uchi et al., 2015; Schellenberg et al., 2018), which consistently show that calculated TF contact force overpredicts the force measured by the prosthesis. It should be recognized that the present knee model has cruciate ligaments, while the GC measured forces are from ligament-sacrificing prostheses that were instrumented and surgically implanted during a TKA procedure. Because cruciate ligaments are capable of transmitting the shear forces across the knee joint to the TF articular surfaces (O'Connor et al., 1990b), our model might be expected to overpredict forces measured from instrumented TKA prostheses.

Finally, the parameter sensitivity analysis performed at 90 degrees of knee flexion (Figure A3) may be useful for identifying the most important variables in the model. For example, the PF and QT forces were most sensitive to the radius of curvature of the PF notch, whereas TF contact forces were most sensitive to the length of the AC ligament and a variety of anatomical measures of the femoral condyle. These findings point to the need for accurate, subject-specific musculoskeletal anatomy to ensure valid force predictions at more extreme knee angles.

### Limitations

While the predicted reductions in knee joint tissue forces with the simulated TCU brace were considerable, they should be considered carefully in light of some of the limitations of the study. For example, we assumed in the simulation that participants were able to adapt muscle effort to produce identical kinematics and kinetics of the lower leg with and without the brace. Support for this assumption is provided by biomechanical studies of experimental exoskeletons that provide an assistive moment to joints of the lower extremity (Kao et al., 2010; Lewis and Ferris, 2011). While these studies did not explore internal joint forces, they do show that humans are quickly able to adapt muscle effort in the presence of an external moment applied to the hip (Lewis and Ferris, 2011) or ankle (Kao et al., 2010) to maintain invariant net joint moments. Therefore, our implicit assumption of invariant net knee moment during the DKB is reasonable for simulating the action of the TCU.

We also assumed the brace has perfect force transmission to the user (i.e., the spring force in the brace is entirely transmitted as a force perpendicular to the tibia) and that the brace does not deflect or become misaligned with the knee axis of rotation. Future studies applying the brace to human subjects directly will require a means of quantifying force transmission at the skin interface and moment arm of the brace(s) to verify the assistive moment being generated.

In general, the knee model overestimated *in vivo* TF forces (Figure 7). The model ignores structures such as collateral ligaments, meniscus, posterior structures (popliteal tendon for example), joint capsule, fascia, and skin. The relative contribution of these structures to sagittal plane joint contact force is likely marginal; nevertheless, ignoring other structures of the knee means that joint forces based on our simple model are probably overestimated. However, the model also ignores co-contraction, so forces may be underpredicted in some circumstances, and these findings may not generalize to OA participants who exhibit elevated co-contraction of leg muscles (Hubley-Kozey et al., 2008).

We interpret our findings as tricompartmental unloading, yet we cannot directly confirm unloading of both medial and lateral condyles of the TF joint with the model we used. The TF contact model is represented in our study by a single set of articulating condyles; therefore, we could only compute the overall joint contact force and not the medial and lateral distribution. It stands to reason, though, that a reduction in knee extensor muscle force would reduce the contact force for both medial and lateral condyles.

A final limitation of this study was the fact that the resulting sample was six females and two males, which prevented any meaningful comparison of biological sex.

## Conclusions

The biomechanical analysis of the simulated TCU brace provides proof-of-concept evidence in support of a TCU knee extension-assist brace to significantly reduce forces for all major knee structures during a DKB. The primary mechanism of unloading was reduction of muscle forces, which reduced forces transmitted to all internal structures of the knee. Three different TCU brace models were simulated, which altered the degree of brace assistance as expected. This suggests the technology can be developed to provide a customized level of assistance, all which should have a benefit in reducing knee forces, which is the intended benefit of the TCU brace.

## Data Availability Statement

Data supporting the conclusions of this article will be made available by the authors, without undue reservation.

## Ethics Statement

The studies involving human participants were reviewed and approved by University of New Brunswick, Research Ethics Board. Written informed consent for participation was not required for this secondary study in accordance with the national legislation and the institutional requirements.

## Author Contributions

All authors contributed to the interpretation of the data and drafting the manuscript. CM wrote the computer code for the biomechanical analysis, acquired the data used in the study and conducted the statistical analysis. EB and CM developed the knee model sensitivity analysis. SB conducted the Grand Challenge analysis. EB and CC-S generated the brace model data. All authors have read and approved the final submitted version.

## Conflict of Interest

CC-S was President and CEO of Spring Loaded Technologies Inc. The remaining authors declare that the research was conducted in the absence of any commercial or financial relationships that could be construed as a potential conflict of interest.
