# Supplementary material for: Biomechanical Study of a Tricompartmental Unloader Brace for Patellofemoral or Multicompartment Knee Osteoarthritis
Source: Front Bioeng Biotechnol. 2021 Jan 28;8:604860. doi: 10.3389/fbioe.2020.604860 (PMC7876241; doi:10.3389/fbioe.2020.604860)
Supplement: Supplementary file 1 [file Presentation_1.pdf]

APPENDIX I

A1. Kinematic Model of the Knee

Relative motion between the thigh and shank segments of the leg can be described in the sagittal plane by the angle formed between the two segments (knee flexion angle) and the position of the femur relative to the tibia. Figure A1 illustrates how the femoro-tibial contact point (*FT*) moves posteriorly on the tibia plateau as the knee flexes. Because the femoral condyle contact length is longer than the tibial condyle contact length, the articular surfaces must roll and slide. For this motion to occur the instant centre of rotation (*IC*) must be located somewhere between the femoral condyle centre of curvature (*OC*) and the contact point *FT*.

A mechanism that satisfies these criteria was first proposed by Kapandji (43) and later expanded by O'Connor and colleagues (27–34). This theory explains the sagittal plane kinematics of the knee in terms of its posterior translation during knee flexion and its stability at the extremes of motion. The model is based on the "isometric fiber theory" which states that the anterior cruciate ligament (ACL) and posterior cruciate ligament (PCL) contain a neutral isometric fibre that forms a crossed four-bar linkage in the sagittal plane. The instant centre of rotation, *IC*, between the femur and tibia is therefore located at the intersection of the PCL and ACL links.

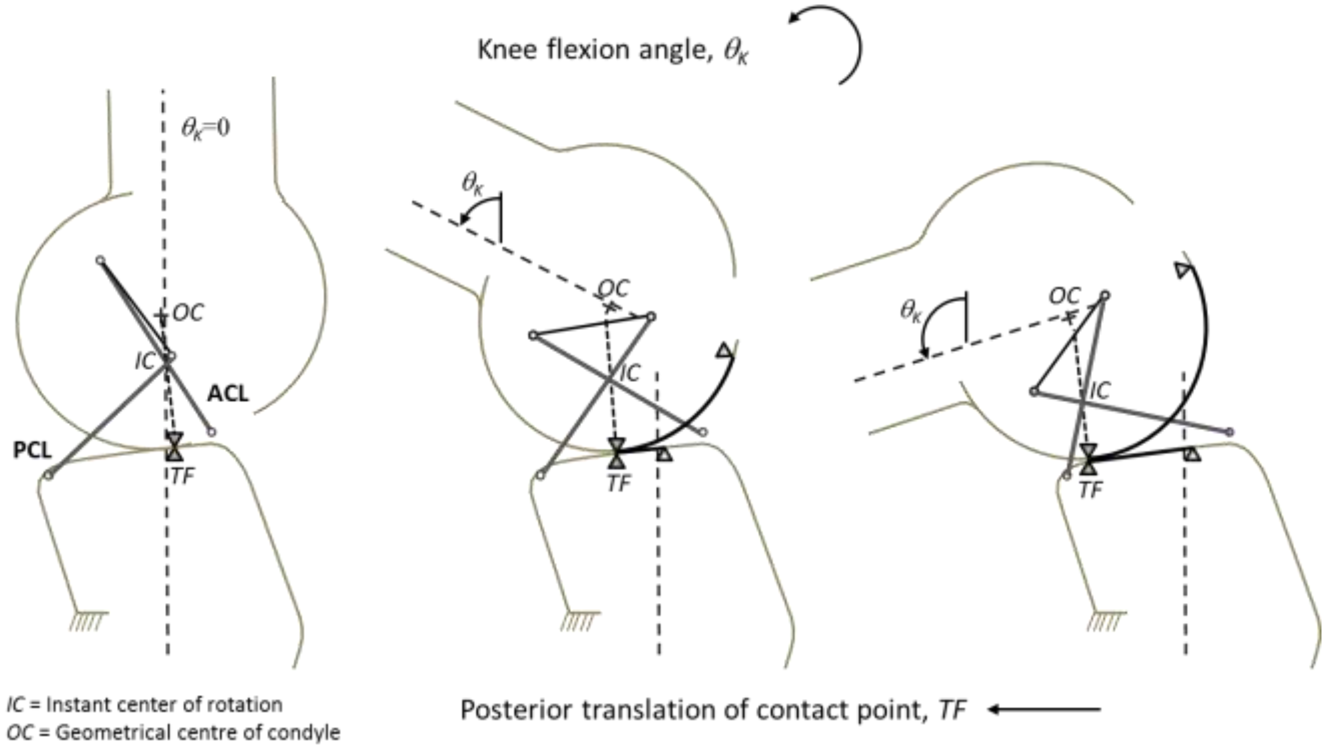

Figure A1. Kinematic model of the knee that defines the pathway of femoro-tibial contact (FTJ) based on the geometry of cruciate ligament (ACL and PCL) complex and knee flexion angle in the sagittal plane (0-130 deg)

A1.1 Cruciate Ligaments and Joint Contact

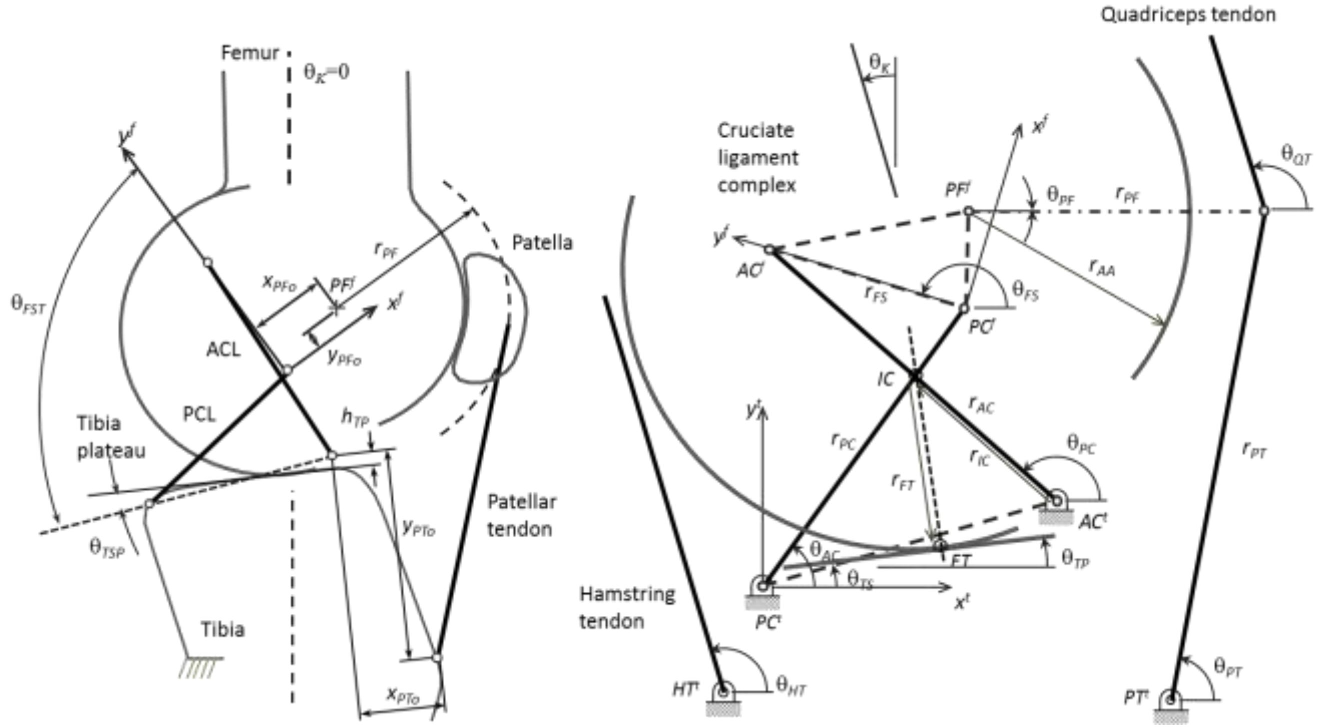

Figure A2. Knee geometry in the sagittal plane from O'Connor et al. 1990 (44).

Figure A2 shows the orientation of the cruciate crossed 4-bar and the knee extensor and flexor mechanisms of the knee as detailed in O'Connor et al. (44). The angle of the fixed tibia link (attachment line) of the cruciate mechanism θ_{TS} is a function of the angle between tibia plateau and tibia attachment line θ_{TSP} and the angle of the tibia plateau θ_{TP} relative to the tibia frame

$$\theta_{TS} = \theta_{TP} + \theta_{TSP} \quad \text{(A1)}$$

At full extension of the knee the ACL isometric fibre runs parallel to the femoral link, as shown in the left side of Figure A2. Therefore the angle of the moving femoral link θ_{FS} is a function of knee flexion angle θ_K and the angle between the femoral attachment and tibia attachment lines of the cruciates, θ_{FST}.

$$\theta_{FS} = \theta_K + (\pi - \theta_{FST}) + \theta_{TS} \quad \text{(A2)}$$

Given the dimensions of the 4-bar consisting the femoral attachment link length (r_{FS}), the tibia attachment link length (r_{TS}), and the lengths of the cruciate ligaments (r_{AC} and r_{PC}), the angles of the ACL (θ_{AC}) and PCL (θ_{PC}) can be calculated from Freudenstein's equation. For the PCL link

$$\theta_{PC} = 2\tan^{-1}\left[\frac{A \pm \sqrt{A^2+B^2-C^2}}{B+C}\right] \quad \text{(A3)}$$

where

$$A = 2r_{PC}(r_{TS}\sin\theta_{TS} - r_{FS}\sin\theta_{FS}) \quad \text{(A4)}$$

$$B = 2r_{PC}(r_{TS}\cos\theta_{TS} - r_{FS}\cos\theta_{FS}) \quad \text{(A5)}$$

$$C = r_{TS}^2 + r_{PC}^2 + r_{FS}^2 - r_{AC}^2 - 2r_{FS}r_{TS}\cos(\theta_{TS} - \theta_{FS}) \quad \text{(A6)}$$

and similarly for the ACL link

$$\theta_{AC} = 2\tan^{-1}\left[\frac{D \pm \sqrt{D^2+E^2-F^2}}{E+F}\right] \quad \text{(A7)}$$

where

$$D = -2r_{AC}(r_{TS}\sin\theta_{TS} - r_{FS}\sin\theta_{FS}) \quad \text{(A8)}$$

$$E = -2r_{AC}(r_{TS}\cos\theta_{TS} - r_{FS}\cos\theta_{FS}) \quad \text{(A9)}$$

$$F = r_{TS}^2 - r_{PC}^2 + r_{FS}^2 + r_{AC}^2 - 2r_{FS}r_{TS}\cos(\theta_{TS} - \theta_{FS}) \quad \text{(A10)}$$

The y-coordinates of the attachment points of the ligaments are defined relative to the distal endpoint (ankle), and the x-coordinate is defined so the tibia long axis passes through the knee instant centre at full extension (44).

$$x_{AC}^t = -(r_{AC} - r_{FS})\cos(\pi - \theta_{FST})\,;\;\; y_{AC}^t = l_{tib} \quad \text{(A11)}$$

$$x_{PC}^t = x_{AC}^t - r_{TS}\cos\theta_{TS}\,;\;\; y_{PC}^t = y_{AC}^t - r_{TS}\sin\theta_{TS} \quad \text{(A12)}$$

where l_{tib} is length of the tibia (from ankle to tibia crest). The position of the instant centre (*IC*) at the intersection of the ACL and PCL “isometric” fibres is then calculated in tibia coordinates from

$$x_{IC}^t = x_{AC}^t + r_{IC}\cos\theta_{AC}\,;\;\; y_{IC}^t = y_{AC}^t + r_{IC}\sin\theta_{AC} \quad \text{(A13)}$$

where r_{IC} is the position of *IC* along the ACL relative to the tibia attachment

$$r_{IC} = r_{TS}\frac{\sin(\theta_{TS}-\theta_{PC})}{\sin(\theta_{PC}-\theta_{AC})} \quad \text{(A14)}$$

The position of femoro-tibial contact (*FT*) is located on the tibia plateau such that its normal vector passes through the joint *IC* position

$$x_{FT}^t = x_{IC}^t + r_{FT}\sin\theta_{TP}\,;\;\; y_{FT}^t = y_{IC}^t - r_{FT}\cos\theta_{TP} \quad \text{(A15)}$$

where r_{FT} is the position of *FT* relative to *IC* along the contact normal.

$$r_{FT} = h_{TP} + r_{TS}\frac{\sin(\theta_{PC}-\theta_{TS})\sin(\theta_{AC}-\theta_{TP})}{\sin(\theta_{AC}-\theta_{PC})} \quad \text{(A16)}$$

A1.2 Extensor and Flexor Mechanism

The coordinates of the attachments of the patellar tendon and hamstring tendon are provided from O'Connor et al. (44) and are referenced to ACL insertion and tibia plateau.

$$x_{PT}^{t} = x_{AC}^{t} + x_{PTo}\cos\theta_{TP} - y_{PTo}\sin\theta_{TP}\ ; \quad y_{PT}^{t} = y_{AC}^{t} + x_{PTo}\sin\theta_{TP} + y_{PTo}\cos\theta_{TP} \qquad \text{(A17)}$$

The angle of the patellar tendon and patella-femoral contact are calculated by treating the knee extensor mechanism as a six-bar linkage where the patella-femoral link has its centre (PF) located on the femur relative to the PCL insertion

$$x_{PF}^{f} = x_{PC}^{f} + x_{PFo}\cos\vartheta - y_{PFo}\sin\vartheta\ ; \ y_{PF}^{f} = y_{PC}^{f} + y_{PFo}\cos\vartheta + x_{PFo}\sin\vartheta \qquad \text{(A18)}$$

where

$$\vartheta = \theta_{FS} - \pi/2 \qquad \text{(A19)}$$

$$x_{PC}^{f} = x_{PC}^{t} + r_{PC}\cos\theta_{PC}\ ; \ y_{PC}^{f} = y_{PC}^{t} + r_{PC}\sin\theta_{PC} \qquad \text{(A20)}$$

The angle of the patellar tendon is found from

$$\theta_{PT} = 2\tan^{-1}\left[\frac{G \pm \sqrt{G^2+H^2-I^2}}{H+I}\right] \qquad \text{(A21)}$$

where

$$G = 2r_{PT}\left(y_{PF}^{f} - y_{PT}^{t}\right) \qquad \text{(A22)}$$

$$H = 2r_{PT}\left(x_{PF}^{f} - x_{PT}^{t}\right) \qquad \text{(A23)}$$

$$I = \left(x_{PF}^{f} - x_{PT}^{t}\right)^2 + \left(y_{PF}^{f} - y_{PT}^{t}\right)^2 + r_{PT}^2 - r_{PF}^2 \qquad \text{(A24)}$$

and the patella-femoral contact angle is found from

$$\theta_{PF} = 2\tan^{-1}\left[\frac{J \pm \sqrt{J^2+K^2-L^2}}{K+L}\right] \qquad \text{(A25)}$$

where

$$J = -2r_{PF}\left(y_{PF}^{f} - y_{PT}^{t}\right) \qquad \text{(A26)}$$

$$K = -2r_{PF}\left(x_{PF}^{f} - x_{PT}^{t}\right) \qquad \text{(A27)}$$

$$L = \left(x_{PF}^{f} - x_{PT}^{t}\right)^2 + \left(y_{PF}^{f} - y_{PT}^{t}\right)^2 - r_{PT}^2 + r_{PF}^2 \qquad \text{(A28)}$$

The quadriceps tendon follows the femur until the tendon contacts and wraps around the anterior arc of the patella-femoral notch.

If $\cos(\theta_K - \theta_{PF}) > r_{AA}/r_{PF}$ then

$$\theta_{QT} = \theta_K + \pi/2 \quad \text{(A29)}$$

If $\cos(\theta_K - \theta_{PF}) \leq r_{AA}/r_{PF}$ then

$$\theta_{QT} = \theta_{PF} + \pi - \sin^{-1}(r_{AA}/r_{PF}) \quad \text{(A30)}$$

Finally the hamstring tendon is simply assumed to follow the femur, with tibia attachments as described by O'Connor et al. (44).

$$\theta_{HT} = \theta_K + \pi/2 \quad \text{(A31)}$$

$$x_{HT}^t = x_{AC}^t + x_{HTo}\cos\theta_{TP} - y_{HTo}\sin\theta_{TP}\ ;$$

$$y_{HT}^t = y_{AC}^t + x_{HTo}\sin\theta_{TP} + y_{HTo}\cos\theta_{TP} \quad \text{(A32)}$$

Input coordinate data, shown in Table A1, are scaled to tibia length

$$s = l_{tib}/48 \quad \text{(A33)}$$

APPENDIX II

II. Force Model of the Knee

Considering the free-body diagram of the tibia (Figure 2, right) where known forces applied to tibia via ankle joint (net ankle force and moment), gravity, and inertia (force and moment) must be balanced by unknown forces in the joint's structures. Assuming the geometry of knee structures is known at any knee flexion angle (using the model above), the forces in at least one muscle tendon, ligament and contact force can be computed to satisfy dynamic equilibrium of the shank. In other words, three equations

$$\Sigma F_x = m_s a_{sx};\ \Sigma F_y = m_s a_{sy};\ \Sigma M_z = I_s \alpha_s \quad \text{(A34)}$$

are used to compute three unknown forces

$$F_{tendon};\ F_{ligament};\ F_{contact} \quad \text{(A35)}$$

that satisfy basic tissue constraints: tensile tendon and ligament forces and compressive contact forces.

Clearly the knee has more than three structures that bear and transmit force through the joint. However, if we ignore co-contraction we can argue that muscle tendon forces will either be in the extensor or flexor tendons, depending on whether the external forces cause a flexion or extension moment. The ACL and PCL are oriented to resist shear forces anteriorly or posteriorly depending on the external force line of action. The contact force, on the other hand, should always exist when the leg bears weight, or when muscles contract, or both.

Therefore we can consider four different models, where one of the two tendons is combined with one of the two cruciate ligaments plus the contact force, as previously described by O'Connor et al. (45). Applying the constraint conditions for each frame (i=1,2,…n) gives logical arrays

$$i_{PAC} = \{F_{PT}>0 \wedge F_{AC}>0 \wedge -F_{FT}>0\} \quad \text{(A36)}$$

$$i_{PPC} = \{F_{PT}>0 \wedge F_{PC}>0 \wedge -F_{FT}>0\} \quad \text{(A37)}$$

$$i_{HAC} = \{F_{HT}>0 \wedge F_{AC}>0 \wedge -F_{FT}>0\} \quad \text{(A38)}$$

$$i_{HPC} = \{F_{HT}>0 \wedge F_{PC}>0 \wedge -F_{FT}>0\} \quad \text{(A39)}$$

which are used to construct the solution[1]

$$F_{tendon(extensor|flexor)} = F_{PT}(i_{PAC}) + F_{PT}(i_{PPC}) \mid F_{HT}(i_{HAC}) + F_{HT}(i_{HPC}) \quad \text{(A40)}$$

$$F_{ligament(ACL|PCL)} = F_{AC}(i_{PAC}) + F_{AC}(i_{HAC}) \mid F_{PC}(i_{PPC}) + F_{PC}(i_{HPC}) \quad \text{(A41)}$$

[1] Note, the solution is illustrated with only one flexor muscle candidate. The gastrocnemius, for example, is also a knee flexor, and could be used to generate another set of candidate solutions, as demonstrated by O'Connor et al [45]. In this case an additional step would be required for selecting the candidate model based on a reasonable criteria, such as the model with the lowest compression force.

$$F_{contact} = F_{FT}(i_{PAC}) + F_{FT}(i_{HAC}) + F_{FT}(i_{PPC}) + F_{FT}(i_{HPC}) \quad \text{(A42)}$$

The general approach for computing tendon, ligament and contact forces is described below. Since there are three unknown forces having known orientation and position, moments are simply summed about the intersection of two of the unknown forces to compute the magnitude of the third force vector. This is repeated for each of the three unknown forces.

Let the external forces (ankle reaction force, gravity, inertia force and brace reaction force, etc.) and moments on the segment (ankle reaction moment and inertia moment, etc.) be defined as a set: $F_{e(f)}$ where f=1,2,…Nf, and $M_{e(m)}$ where m=1,2,…Nm, respectively. The moment centre for F_k is the intersection of lines of action of F_i and F_j, or c_{ij}, and so on for the other two moment centres.

$$c_{ij} = \frac{F_i}{\|F_i\|} \cap \frac{F_j}{\|F_j\|};\ c_{ik} = \frac{F_i}{\|F_i\|} \cap \frac{F_k}{\|F_k\|};\ c_{jk} = \frac{F_j}{\|F_j\|} \cap \frac{F_k}{\|F_k\|} \quad \text{(A43-45)}$$

The moment arm r_k is the perpendicular distance between c_{ij} and line of action of F_k, and so on for the other two moment arms.

$$r_k = \|c_{ij} \perp F_k\|;\ r_j = \|c_{ik} \perp F_j\|;\ r_i = \|c_{jk} \perp F_i\| \quad \text{(A46-48)}$$

The moment arms for each external force can also be found

$$r_{e(f)}^{ij} = \|c_{ij} \perp F_{e(f)}\|;\ r_{e(f)}^{ik} = \|c_{ik} \perp F_{e(f)}\|;\ r_{e(f)}^{jk} = \|c_{jk} \perp F_{e(f)}\| \quad \text{(A49-51)}$$

Which allows independent calculation of the three unknown forces

$$F_k = -\frac{\sum_{f=1}^{Nf} r_{e(f)}^{ij} \times F_{e(f)} + \sum_{m=1}^{Nm} M_{e(m)}}{r_k} \quad \text{(A52)}$$

$$F_j = -\frac{\sum_{f=1}^{Nf} r_{e(f)}^{ik} \times F_{e(f)} + \sum_{m=1}^{Nm} M_{e(m)}}{r_j} \quad \text{(A53)}$$

$$F_i = -\frac{\sum_{f=1}^{Nf} r_{e(f)}^{jk} \times F_{e(f)} + \sum_{m=1}^{Nm} M_{e(m)}}{r_i} \quad \text{(A54)}$$

Finally, once the patellar tendon force F_{PT} is calculated (and found to be >0) the patella-femoral contact force F_{PF} and quadriceps tendon force F_{QT} are easily found by treating the patella as a 3-force body. Given the known angles of the force components, θ_{PT}, θ_{PF} and θ_{QT} (equations A21, A25, and A29-30, respectively), unknown forces F_{PT} and F_{PF} can be found.

$$F_{PF} = -F_{PT}\frac{\sin(\theta_{QT}-\theta'_{PT})}{\sin(\theta_{QT}-\theta_{PF})} \quad \text{(A55)}$$

$$F_{QT} = -F_{PT}\frac{\sin(\theta_{PF}-\theta'_{PT})}{\sin(\theta_{PF}-\theta_{QT})} \quad \text{(A56)}$$

where $$\theta'_{PT} = \pi - \theta_{PT} \quad \text{(A57)}$$

APPENDIX III

Figure A3 shows total uncertainty for each loaded structure (at 90 degrees knee flexion) by stacked bar charts, composed of relative contributions from each input parameter (see Table A1) using equations 2-3. Overall the calculated cumulative uncertainties were relatively small (ranging between .1BW to .35BW). Uncertainty in radius of the PF notch (r_{AA}) had notable effects on QT and PF forces, while uncertainties in ACL length (r_{AC}), length of the PF link (r_{PF}), y-coordinate of centre of curvature of the PF notch (y_{PF}), and femoral cruciate attachment points (r_{FS}) were most influential for computing TF and PC forces.

Table A1. Input parameters of the knee model

Description of Parameter	Symbol	Value
Length of tibial attachment link, TS	r_{TS}	s*3.05 cm
Length of PCL	r_{PC}	s*3.22 cm
Length of femoral attachment link. FS	r_{FS}	s*1.28 cm
Length of ACL	r_{AC}	s*2.99 cm
Angle of TS relative to tibia plateau	θ_{TSP}	13.0 deg
Angle of FS relative to TS at full extension	θ_{FST}	80.0 deg
Angle tibia plateau relative to tibia long axis normal	θ_{TP}	7 deg
Offset of tibia plateau to ACL insertion	h_{TP}	s*0.6 cm
Length of patellar tendon (PT) link	r_{PT}	s*8.0 cm
Length of patello-femoral (PF) link	r_{PF}	s*4.6 cm
Radius of patello-femoral notch	r_{AA}	s*2.3 cm
PT insertion on tibia relative to ACL insertion and tibia plateau	x_{PT},y_{PT}	s*(2.2, -4.4) cm
Center of anterior arc relative to the PCL insertion and femoral attachment line	x_{PF},y_{PF}	s*(2.0, 0.5) cm
Hamstring tendon insertion on tibia relative to ACL insertion and tibia plateau	x_{HT},y_{HT}	s*(-4.5, -1.8) cm

s = scale factor (1.0 for 48cm tibia)

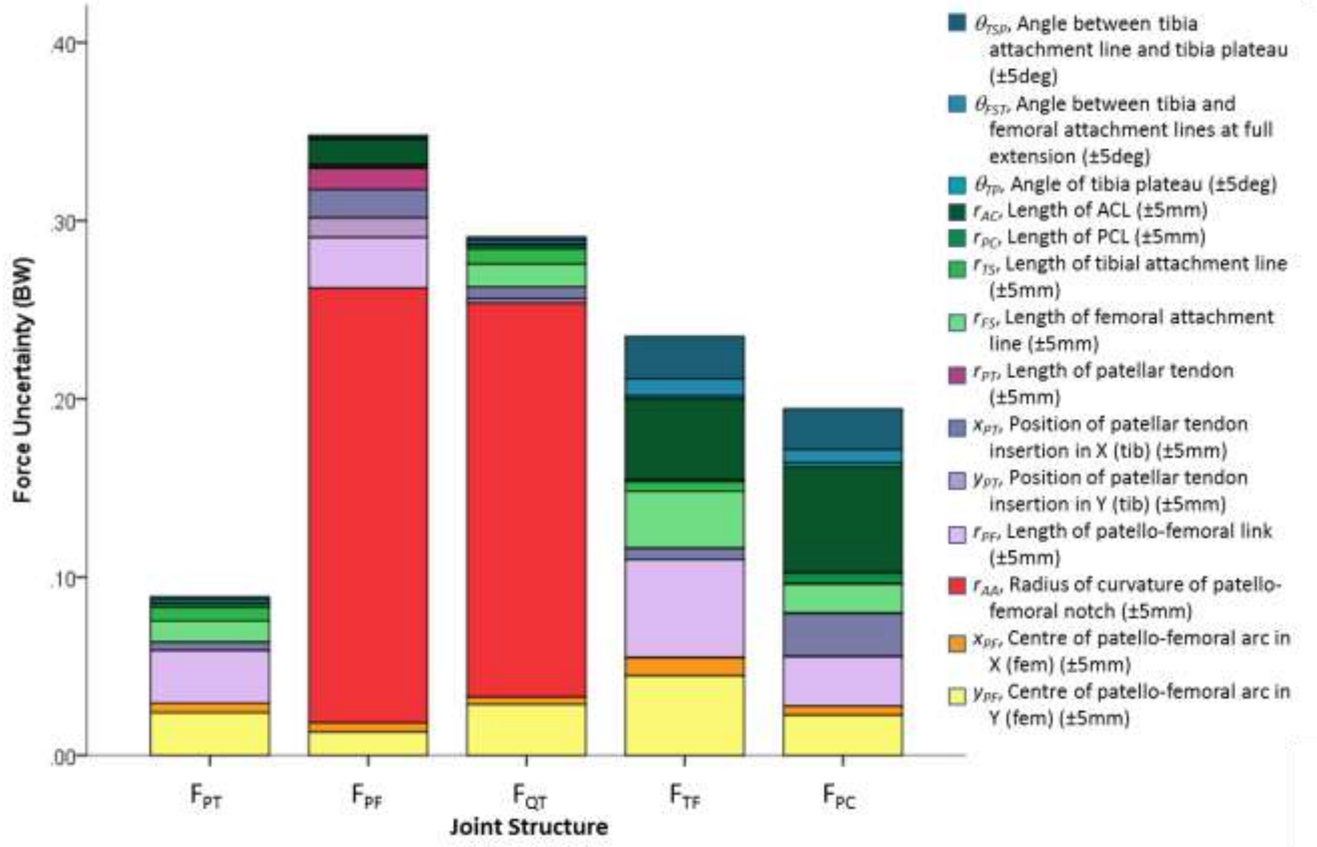

Figure A3. Sensitivity Analysis: Injecting +/- 5 mm and +/- 5 deg uncertainty in linear angular dimensions and combining force output uncertainty using an expanded Taylor's series. Stacked bars show relative contributions of each model parameter. Column height represents the total expected uncertainty in force.
